# Histopathologic features of nutritional deficiency dermatoses: A two-center series

**DOI:** 10.1016/j.jdin.2025.10.018

**Published:** 2025-11-20

**Authors:** Silvija Milanovic, Emily Draper, Milbrey Parke, Catherine G. Chung, Abena Minta, Kiran Motaparthi

**Affiliations:** a University of Florida, College of Medicine, Gainesville, Florida; b Department of Dermatology, University of Florida College of Medicine, Gainesville, Florida; c Department of Pathology, The Ohio State University Wexner Medical Center, Columbus, Ohio; d Department of Dermatology, The Ohio State University Wexner Medical Center, Columbus, Ohio

**Keywords:** dermatopathology, histopathology, micronutrients, nutritional, nutritional deficiency, parakeratosis, skin biopsy, skin diseases, zinc deficiency

*To the Editor:* The prevalence of nutritional deficiency dermatoses (NDDs) is rising, likely due to greater awareness and earlier dermatologic consultation.^1^ Prolonged hospitalization places critically ill patients at risk for both macro- and micro-nutrient deficiencies, which often present with subtle cutaneous findings (Fig 1, *A* and *B*).^2^ While certain histopathologic features of NDDs have become familiar in dermatopathology, they are largely based on limited case reports.^1^^,^^2^ In this retrospective review from 2 tertiary care centers, we examine associations between micronutrient deficiencies and histopathologic findings.

**Fig 1:**
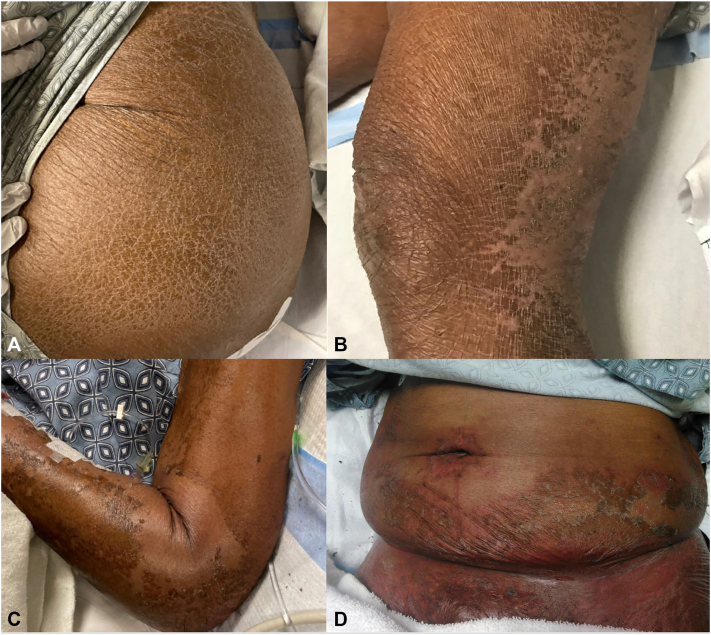
**A,** Kwashiorkor: Asteatotic-like dermatitis affecting the buttock. **B,** Kwashiorkor: Asteatotic-like dermatitis with dyspigmentation and a flaky paint appearance affecting the lower extremity. **C,** Kwashiorkor: Superficial desquamation, erythema, and flaky paint appearance of the forearm. **D,** Kwashiorkor: Erythema with superficial desquamation and flaky paint appearance on the lower abdomen.

Patients from the University of Florida and Ohio State University were included. Their inpatient dermatology consult databases were queried for NDD cases from January 1, 2015, to February 1, 2022. The diagnosis of nutritional deficiency was established based on clinical findings and laboratory evaluation; all patients had physical exam findings (Fig 1, *A*-*D*) consistent with NDDs that were confirmed by laboratory evidence of macronutrient and/or micronutrient deficiency. A total of 29 biopsies from 24 patients were reviewed. Two dermatopathologists independently assessed histopathologic features, with discrepancies resolved by consensus. Continuous and categorical variables were analyzed using Wilcoxon-Mann-Whitney and Fisher’s exact tests, respectively.

Associations between clinical variables (age, sex, race, levels of vitamins A, B1, B6, C, and E, zinc, albumin, and total protein; Supplementary Table I, available via Mendeley at https://data.mendeley.com/datasets/3gsmsshgbw/4) and histopathologic variables (Supplementary Table II, available via Mendeley at https://data.mendeley.com/datasets/3gsmsshgbw/4) were investigated. Demographic factors were not significantly associated with specific histopathologic features. Parakeratosis (Fig 1, *A* and *B*) and loss of the granular layer were the most common features, present in 93.1% of biopsies. Another common feature was dermal edema (Fig 2, *B*), present in 72.4% of biopsies. The most infrequently described histopathologic features were pallor (17.2%) and ballooning degeneration (17.2%). In terms of laboratory values, pallor was associated with lower total protein (4.82 ± 0.81 vs 6.33 ± 1.71, *P* = .0234). Low vitamin, zinc, and albumin levels were not significantly associated with any histopathologic changes. Previously described histopathologic features of NDD include psoriasiform epidermal hyperplasia and confluent parakeratosis (Fig 2, *C* and *D*); keratinocyte pallor (Fig 2, *D*) is considered a classic or pathognomonic finding.^3^ However, most biopsies in our series lacked these features and instead showed nonspecific or asteatotic dermatitis-like changes (ie, compact hyperkeratosis without spongiosis) (Fig 2, *A* and *B*), underscoring the limitations of histopathology alone. Epidermal pallor, emphasized in early descriptions of acrodermatitis enteropathica, may reflect early-stage disease.^4^ In later stages, pallor often disappears while confluent parakeratosis persists.^5^ In our cohort, pallor was rare (17%), typically subtle, and most often observed in younger patients (average age 31), supporting its association with earlier presentation.^4^ Confluent parakeratosis, though nonspecific, was more frequent (72%) and may serve as a more sensitive histopathologic indicator of NDD.

**Fig 2:**
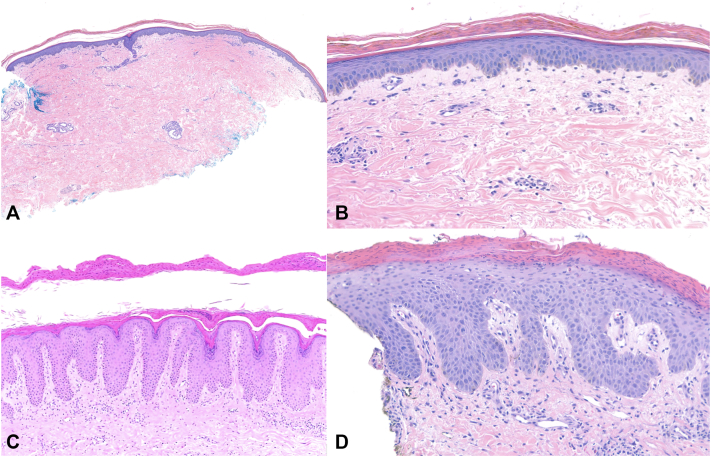
**A,** Kwashiorkor with asteatotic dermatitis-like features. Subtle features of nutritional deficiency include slight epidermal atrophy and parakeratosis (H&E, 50× magnification). **B,** Kwashiorkor with asteatotic dermatitis-like features. Subtle features of nutritional deficiency include confluent parakeratosis, a retained granular layer, slight epidermal atrophy, and dermal edema (H&E, 200× magnification). **C,** Psoriasiform epidermal hyperplasia with areas of hypogranulosis and overlying confluent parakeratosis (H&E, 100× magnification). **D,** Classic or well-described features of nutritional deficiency include a psoriasiform appearance owing to regular hyperplasia, confluent parakeratosis, and sparse dermal inflammation. A diminished granular layer, pallor of the spinous layer, dysmaturation, dyskeratosis, and papillary dermal edema are also observed (H&E, 200× magnification).

Among histopathologic features, only epidermal pallor was significantly associated with lower total protein levels. This may reflect impaired keratinocyte maturation in protein malnutrition, where hypoproteinemia disrupts synthesis of structural proteins and enzymes essential for normal epidermal differentiation.^5^

While classic clinical (Fig 1, *C* and *D*) and histopathologic features (Fig 2, *D*) of NDD are well described, nonclassic findings remain nonspecific and underrecognized.^3^ As a result, micronutrient deficiencies may be overlooked when diagnosis relies heavily on biopsy. These findings underscore the importance of integrating clinical history, lab data, and histopathology. The main limitation of this study is the small sample size within each deficiency subgroup, which limits statistical power. This study lays the groundwork for future, larger-scale investigations.

### Declaration of generative AI and AI-assisted technologies in the writing process

No AI tools were used in the data analysis, figure generation, or manuscript content. All intellectual and analytical contributions remain those of the authors.

## Conflicts of interest

None disclosed.

## References

[1] Kroshinsky D., Cotliar J., Hughey L.C., Shinkai K., Fox L.P. (2016). Association of dermatology consultation with accuracy of cutaneous disorder diagnoses in hospitalized patients: a multicenter analysis. JAMA Dermatol.

[2] Kuhl J., Davis M.D.P., Kalaaji A.N., Kamath P.S., Hand J.L., Peine C.J. (2004). Skin signs as the presenting manifestation of severe nutritional deficiency: report of 2 cases. Arch Dermatol.

[3] Garcia D., Nielson C.B., Gillihan R., Schoch J., Auerbach J., Motaparthi K. (2020). Xerotic eruption and purpura: answer. Am J Dermatopathol.

[4] Gonzalez J.R., Botet M.V., Sanchez J.L. (1982). The histopathology of acrodermatitis enteropathica. Am J Dermatopathol.

[5] Sugiyama A., Fujita Y., Kobayashi T. (2011). Effect of protein malnutrition on the skin epidermis of hairless mice. J Vet Med Sci.

